# Termination of Ca^2+^ Release for Clustered IP_3_R Channels

**DOI:** 10.1371/journal.pcbi.1002485

**Published:** 2012-05-31

**Authors:** Sten Rüdiger, Peter Jung, Jian-Wei Shuai

**Affiliations:** 1 Institut für Physik, Humboldt-Universität zu Berlin, Berlin, Germany; 2 Department of Physics and Astronomy, Ohio University, Athens, Ohio, United States of America; 3 Department of Physics and Institute of Theoretical Physics and Astrophysics, Xiamen University, Xiamen, China; Princeton University, United States of America

## Abstract

In many cell types, release of calcium ions is controlled by inositol 1,4,5-trisphosphate (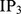) receptor channels. Elevations in 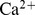 concentration after intracellular release through 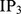 receptors (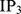
R) can either propagate in the form of waves spreading through the entire cell or produce spatially localized puffs. The appearance of waves and puffs is thought to implicate random initial openings of one or a few channels and subsequent activation of neighboring channels because of an “autocatalytic” feedback. It is much less clear, however, what determines the further time course of release, particularly since the lifetime is very different for waves (several seconds) and puffs (around 100 ms). Here we study the lifetime of 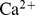 signals and their dependence on residual 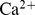 microdomains. Our general idea is that 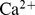 microdomains are dynamical and mediate the effect of other physiological processes. Specifically, we focus on the mechanism by which 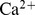 binding proteins (buffers) alter the lifetime of 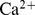 signals. We use stochastic simulations of channel gating coupled to a coarse-grained description for the 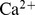 concentration. To describe the 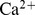 concentration in a phenomenological way, we here introduce a differential equation, which reflects the buffer characteristics by a few effective parameters. This non-stationary model for microdomains gives deep insight into the dynamical differences between puffs and waves. It provides a novel explanation for the different lifetimes of puffs and waves and suggests that puffs are terminated by 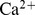 inhibition while 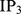 unbinding is responsible for termination of waves. Thus our analysis hints at an additional role of 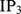 and shows how cells can make use of the full complexity in 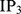
R gating behavior to achieve different signals.

## Author Summary

Calcium signals are important for a host of cellular processes such as neurotransmitter release, cell contraction and gene expression. While the principles of activation and spreading of calcium signals have been largely understood, it is much less clear how their spatio-temporal appearance is shaped. This issue is of high relevance since the spatio-temporal signature is thought to carry the information content. In our paper we study the dynamical mechanisms that determine the time course of calcium release from  receptor channels. We use a stochastic channel description combined with a recently developed model for the distribution of released calcium in a microdomain. The simulations uncover a complex control mechanism, which allows for the tuning of release from short frequent puffs to extended and less frequent wave-like release. Unexpectedly, the model predicts that for wave-like release the dissociation of  from the receptors leads to termination of the calcium signal. This effect relies on a well-known gating property of R channels, which earlier has been regarded as superfluous in studies for groups of channels. Our results also provide a missing link to understand cellular  response to calcium-binding proteins and present a novel mechanism for information processing by R channels.

## Introduction


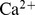
 is a universal messenger of eukaryotic cells and regulates various cellular processes such as morphology, enzyme activity, and gene expression [1], [2]. In many cells, 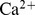 signaling is achieved by modulation of cytosolic 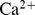 concentrations arising from exchange of 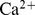 with the endoplasmic reticulum (ER). SERCA pumps produce steep concentration gradients across the ER membrane by moving 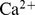 from the cytosol into the ER. Liberation of 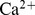 from the ER occurs through inositol 1,4,5-trisphosphate receptor ( 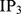
R ) channels in the ER membrane.


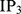
Rs regulate 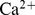 transport in response to binding of 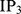 and 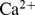 to receptor sites on the cytosolic domain of channels [3]. While binding of the second messenger 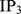 generally promotes the opening of channels, the dependence on cytosolic [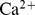] is biphasic. Small increases in [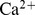] compared to rest level concentrations increase the open probability of 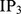
R channels. The dissociation constant, 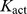, of the responsible binding site is at sub-µM scale. This stimulating 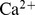 binding gives rise to a self-amplifying mechanism called 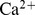 induced 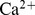 release (CICR): 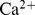 released by one or several channels diffuses in the cytosol and increases the open probability of neighboring channels by binding to their activating binding sites. As the level of 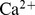 rises further, inhibitory binding of 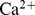 dominates. Consequently, the open probability decreases significantly as 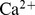 levels reach values comparable to an inhibitory dissociation constant, 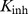, at several tens of µM . Together, the activating and inhibiting binding processes allow for cooperative openings and closings of receptor channels.

Elevations of 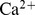 concentrations appear in two basic patterns that reflect the spatial organization of 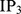
R channels [4]. In many cells, 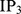
R channels are distributed in clusters on the ER membrane. It is often found that CICR synchronizes channels within clusters, resulting in patterns of localized and short-lived events called puffs [5]. In this regime, 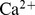 release does not spread to neighboring clusters, which are typically separated by a few µm . Recent studies emphasize the role of sub-cellular 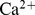 rises for physiological function [6]–[8].

In a different patterning mode, activity of channels from many clusters synchronizes to form cell-wide oscillations [9], [10] or, in larger cells, waves [11]. Global release, lasting for up to several tens of seconds, is triggered by random initiation events (*i.e.*, random openings of a few channels) and carried through the cytosol by CICR between nearby clusters. It is important to note that waves do not only last longer because of the long time it takes to travel through the cell. After a wave has passed, elevations in [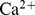] persist for several seconds. In marked contrast, the calcium signal during a puff vanishes within around 100 ms [12]. This discrepancy and the related question how calcium signals are terminated provide the background for this publication.

**Figure 1:**
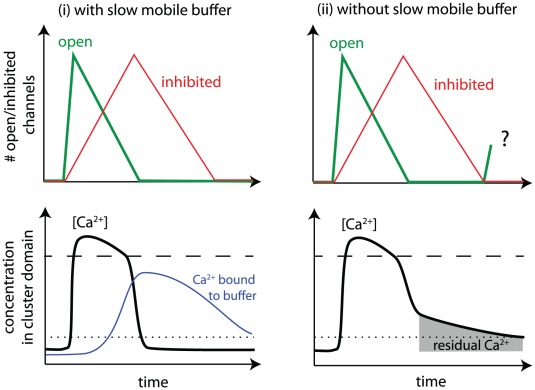
Sketch of single cluster dynamics in presence of slow mobile buffer such as EGTA (i) and in its absence (ii). Collective openings of channels (green lines, top panel) cause a rise in [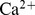] (thick black lines) as well as subsequent slower inhibition of channels (red lines, top panel). Dotted and dashed lines in bottom panel provide comparison of 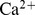 concentration with dissociation constants for activation, 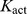, and inhibition, 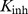, respectively. Free 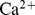 remains in the cluster microdomain transiently (right bottom panel), but its concentration can be reduced below 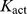 by buffer that binds residual 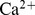 (blue line, left bottom panel). We here ask whether residual 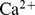 leads to re-openings and, if so, what terminates release despite the re-openings.

Because of their spatial localization, puffs have been studied with theoretical methods by considering a single cluster and ignoring the coupling to channels outside of the cluster. Recently, we and other groups have modeled puffs by a stochastic gating scheme for clustered channels coupled to evolution equations for local 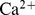 concentration within the cluster microdomain [13]–[18]. The principal dynamics during a puff is sketched in the left panels of Fig. 1 and can be understood qualitatively by comparison of 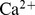 levels with the dissociation constants 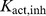. In an initial phase, random opening of a single channel in the cluster drives local [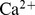] to values above the dissociation constant for activation: [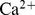] 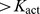 (dotted line). Fast intra-cluster CICR then causes further channels to open (left top panel, green line). Subsequently, 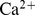 concentration within the cluster microdomain increases strongly. The local 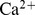 concentration reaches values above the dissociation constant of inhibiting binding sites: [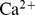]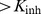 (left bottom panel, dashed line), which leads to inhibition (red line) and eventually to closing of channels.

Since the kinetic rates of the inhibitory sites of 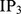
Rs are large, the duration of puffs is short, matching the experimental observations [17]. Furthermore, the modeling predicts that subsequently channels loose 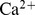 from the inhibition sites rapidly, usually within a time scale comparable to the puff duration. Thus the refractory time after a puff is normally small and channels are available for re-opening shortly after a puff ends [19].

Despite the impressive agreement of this modeling picture with experimental findings, the shortness of the refractory period raises an important question: If channels are inhibited only for tens or hundreds of milliseconds, calcium ions that have been released during the previous puff could re-open the channels shortly by binding again to activating binding sites. The residual [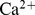] , caused by the 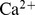 transiently remaining in the microdomain after the last open channel closes, could then be sufficiently large to re-open channels (Fig. 1, right panel). If such re-openings occur repeatedly over an extended period, release may last much longer than the experimentally measured duration of puffs. Using a standard model for 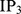 gating coupled to a simple law for [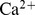] evolution, we first show in this paper that indeed large residual 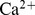 microdomains can generate release at a long time-scale. Because the duration of those events reflects the temporal evolution in the wave regime, from here on we refer to this dynamical regime as waves.

The 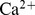 microdomains and the dynamical evolution of residual [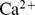] after channel closing was recently studied by us with numerical simulations of the detailed reaction-diffusion equations [20]. Importantly, we found that 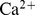 levels can be decaying much slower than previously assumed. This result suggests that residual [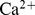] and the mechanism we describe are relevant for the dynamics of 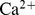 waves. This also implies that residual 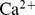 could be a decisive factor for the observation of waves or puffs under different physiological conditions. In this paper we focus on the puff-wave transition under physiological changes related to 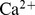 binding proteins.

It is well-known that 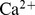 microdomains are very sensitive to certain mobile 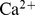 binding and buffering proteins. The buffer proteins alter the distribution of 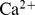 released from channels by reducing the spatial extent of microdomains [21]. Often this leads to strong effects on the dynamics of 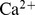 release [12], [21]. However, it was also found that a change of the microdomain extent is small for those buffers that bind 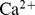 slowly. It therefore was a surprise that slow buffers such as EGTA and parvalbumin can dramatically change the appearance of 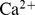 release. While for normal conditions, experiments under stimulating levels of 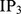 show a wave regime, in presence of slow buffers waves dissolve and puffs appear [12], [22]. To explain this behavior we will here address a lesser known aspect of mobile buffers, which is that the *temporal* relaxation response of the microdomain ( *i.e.*, the residual 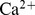 ) is affected.

Our simulations in [20] have shown that binding of 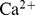 to slow buffers indeed lowers the amplitude of residual 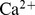 . This effect can be substantial even though the effect on the 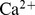 evolution during the puff is small (Fig. 1, left bottom panel). This result suggests that the short duration of puffs, under buffered conditions, is caused by the fast decay of residual 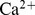 concentrations. Here we therefore propose that the appearance of puffs in cells loaded with EGTA can be understood from the fact that residual 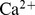 amplitudes are driven close to rest levels by binding to the buffer. Relating the action of buffer to a parameter of our [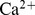] model, we show that indeed the modulation of 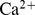 microdomain collapse can explain the puff-wave transition under control of buffer concentration.

This effect entails a final question that we would like to address here: If residual 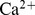 causes sustained channel openings during waves, what then terminates the waves? While we find that puffs are terminated by 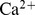 inhibition [13], we also find that the unbinding of 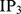 is responsible for termination of waves. Thus 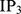 plays a role not only in stimulation of 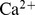 signals and in modulating their amplitude, but dynamics of 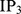 is also important for the termination of 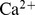 waves. Taken together, this means that wave-like release requires binding of 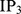 to the receptor for stimulation, slow decay of residual 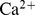 for sustaining the activity for several seconds, and finally unbinding of 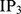 for termination of the wave. Interestingly, the dynamical 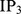 binding/unbinding mechanism that we describe rests on the sensitivity of 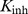 to 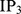 [3]. This sensitivity is a well-established property of the 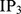 gating to the receptor, but was hitherto neglected in theoretical and numerical studies of 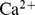 release.

## Model

### Single channel gating

**Figure 2:**
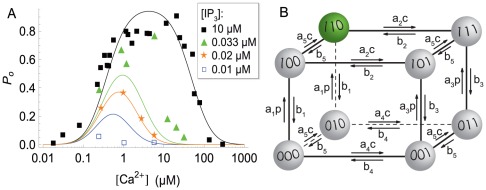
(A) Open probability for various values of cytosolic >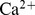 and 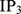 concentrations. Symbols are experimental results from [28], [29], while lines are fitted using DeYoung-Keizer-type models. For increased 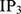 concentrations, curves shift upwards and the maximum shifts to the right. (B) DYK type models can be represented as transitions of each subunit along the edges of a cube. Rates of transitions involving binding of a molecule are proportional to the concentration of the respective molecule (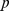 - 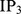 concentration, 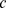 - 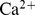 concentration). The factors 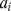, 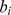 denote the on- and off-rates of binding of ligands to the respective sites.

Equilibrium gating behavior of single channels has been investigated experimentally by patch-clamping [3], [23]. Briefly, experimental results are the following: At small cytosolic 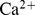 concentrations, an increase in [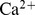] increases the probability of opening, 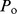. For larger 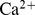 concentrations, an increase in [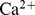] leads to decreases in the open probability (Fig. 2A). Furthermore, binding of the second messenger 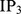 to the receptor increases the open probability. Fig. 2A shows that for higher levels of 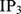 the 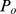 curves are shifted upwards.

Several models have been proposed to discuss the open/close dynamics of a single 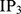
R channel [24], [25]. Here we invoke the DeYoung-Keizer (DYK) model [24], which comprises the three basic binding processes of the receptor in its standard form. An 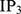
R consists of four identical subunits, each containing three binding sites: an activating site for 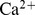 , an inhibiting 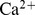 site, and an 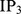 binding site. The three binding sites allow for 8 different states for each subunit, which can be mapped onto triples (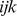) of each subunit. The index 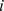 indicates the state of the IP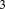 site, 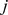 the one of the activating 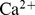 site and 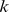 the state of the inhibiting 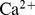 site. An index is 1 if a 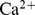 ion or 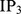 is bound and 0 if not. Following earlier work we require at least three of the four subunits to be in the state (110) for the channel to open [26].

The 8 states allow for 24 different transitions, which can be associated with the edges of a cube (see Fig. 2B). Transitions between the respective subunit states are governed by rate constants, some of which are concentration dependent. There are five pairs of binding/unbinding rates, one for activation and two for inhibition and 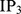 binding, respectively. Each pair is given by the binding rate 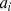 and the unbinding rate 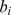. The ratio, 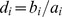, is the dissociation constant, 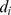. In Fig. 2B, 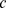 denotes the 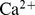 concentration and 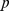 denotes the 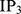 concentration in the cytosol.

We now discuss the properties of the 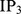
R channel model where we have fitted parameters 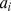 and 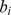 to experimental data (see Supporting Text S1). Solid lines in Fig. 2A show the open probability for a DYK model [27] where parameters were fitted against nuclear patch clamp data taken from [28], [29].

The bell-like shape of the open probability curve is reflected in the different dissociation constants for activation (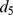) and inhibition (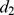 and 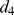). Specifically, the open probability will be large for [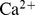] above 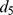 and below 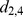. Furthermore, in experiments the exact position of the peak open probability depends on the 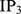 concentration. Often, in the discussion of dynamical models, the 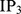 binding process is neglected, or it is absorbed into the activation and inhibition processes by assuming quasi-equilibrium.

The fact that for increasing 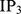 concentration the location of the right tail of the bell curve is sensitive to [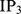] is reflected in the DYK model by the existence of two dissociation constants for inhibition. Accordingly, 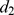, which is the dissociation constant for inhibition at large [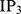] , should be much larger than 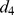, which is the dissociation constant at small [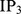] . Indeed, after fitting the DYK model we use 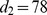 µM while 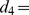
0.111 µM . In contrast, the activation threshold, quantified by 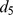, appears to remain constant (in our estimate at 0.25 µM , see Supporting Text S1).

Note that the 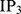 dependence of 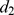 and 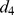 also imposes a corresponding dependence of 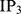 affinity on the binding state of the inhibiting binding site. For all binding/unbinding loops given in Fig. 2B, the thermodynamic constraint requires that in equilibrium the dissociation constants satisfy the detailed balance conditions. Clearly, for a loop involving the activation process, the scheme shown in Fig. 2B is satisfying this condition. However, for a loop involving inhibition and 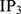 binding we obtain:
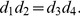
(1)Thus it follows that the 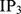 dependence of inhibition requires 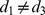, *i.e.*, the 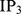 binding depends on the inhibiting site. Because of 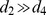, we then have 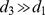. The fact that 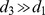 will play a crucial role in the interpretation of our results below.

### Clusters of 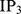
R channels and inhomogeneous 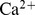 distribution

A major factor in the shaping of 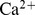 signals is the clustered distribution of 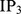 channels on the membrane of the ER. Fluorescence visualization has shown that 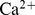 is released through clusters of channels that comprise up to a few tens of channels. Early experimental studies showed that clusters occupy domains of much less than one micrometer in diameter but could not resolve their inner structure. This lead to a general model of a cluster as a small patch on the ER membrane, in which channels are distributed homogeneously and tightly packed. If channels in the cluster open, released calcium creates a *microdomain*, which is characterized by a large calcium concentration and, in this modeling picture, an approximately homogeneous distribution of calcium within the area. Subsequent progress in visualization, however, called this virtual domain picture into question [30] and prompted the inhomogeneous modeling which we will adapt in this study. A further strong hint on the inhomogeneous distribution of 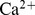 within the cluster originated from our detailed comparison of stochastic simulations with experimental puff data from Smith and Parker [5]. If one assumes a homogeneous distribution of calcium within the cluster microdomain, as was often done in modeling approaches, the experimental amplitude distribution and lifetime of puffs cannot be fitted correctly. However, under the assumption of a non-homogeneous distribution of calcium, we were able to obtain a very accurate fitting of experimental data [17]. Here we further follow recent experimental results in assuming the typical peak open numbers to be 5–10 channels [31].

**Figure 3:**
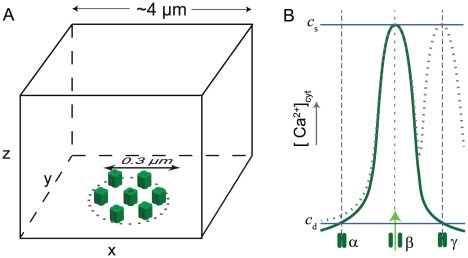
(A) Sketch of the basic setup of our model of 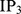
R clusters. Receptors are located on the ER membrane, which is represented by the bottom side of the box. The inside of the box, corresponding to cytosolic space, covers a domain of several µm in each dimension. It models the spatial domain around each cluster. This space provides the surrounding into which released 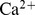 diffuses and does not contain any further 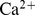 sources. The diameter of clusters is of the order of hundreds of nm (out of scale in the sketch). (B) illustrates our understanding of 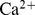 distribution after opening of channels. For simplification three channels located on a row are considered. Numerical simulations have shown that 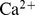 is distributed very inhomogeneously within the cluster microdomain [20]. If a single channel is open (denoted by 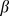), release generates a three-dimensional distribution, which is locally peaked at the open channel and strongly decays away from the channel (blue curve). Thus there are two different scales of [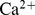] as far as feedback of released 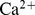 onto the channels is concerned: The first is large at hundreds at µM (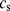 in the figure). It represents the feedback of the released 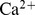 onto the open channel. Much smaller [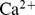] occurs at the location of closed channels (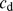 at order of 10 µM ). If two or more channels are open, 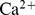 levels increase as illustrated by the dotted line for open channels 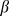 and 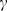. For simplification, 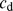 is here taken to be independent on the distance of channels within the cluster. Description by 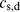 provides a powerful reduction of the complexity of 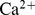 distribution. The approach can be likened to a coarse-graining but with keeping the feedback separated at two scales.

In our paper [20] we numerically simulated detailed models for 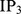
R clusters. Three-dimensional reaction-diffusion equations were used to calculate the evolution of [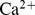] as well as that of relevant buffer proteins. These equations were coupled to a stochastic algorithm for the gating state of each channel. 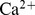 influx through open channels was modeled by disk-shaped two-dimensional source areas on the surface of the simulation domain (see Fig. 3A). The radius of each channel's source area was set to 6 nm. The flux through the source area was adjusted to a current of 0.1 pA per channel in the open state. The size of the simulation domain was large enough (at the order of several µm ) so that the released 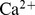 does not significantly alter global 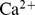 concentrations far away from the open channels. Channels were placed in regular lattices on the domain surface with distance on the order of several tens to hundreds of nm. A main result was that 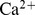 is distributed very inhomogeneously in the cluster microdomain even for small distance of channels. While the concentration directly at the open pore is larger than 100 µM , we found much smaller concentrations in the space between channels, even if measured directly at the membrane surface between two open channels. We concluded that the influence that an open channels exerts to closed channels occurs at a much smaller 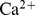 concentration than the feedback that the released 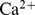 exerts to its own source channel (see Fig. 3B).

To reduce the complexity of this direct numerical approach we introduced a concept of scale separation. While the local concentration at the pore of open channels defines a relatively fixed and large value (denoted 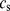 hereafter), the “coupling” concentration, 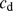, at any closed channel in the cluster is a much smaller quantity (Fig. 3B). In an approximation step we replaced this quantity by a suitable 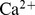 average concentration for the microdomain [17], [20], which is independent on the position of the closed and open channels within the cluster. Our simulations also showed that this quantity, which describes a typical concentration at closed channels within the microdomain, strongly depends on the number of open channels, 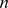. Therefore, and taking into account a small rest level concentration 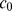, we obtain:
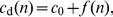
(2)where 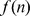 is a function of the number of open channels with 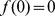. This ansatz assumes that the [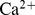] at any closed channel in the cluster follows the number of 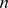 open channels in a quasi-stationary way. In fact, earlier simulations have shown that local increases in the concentration quickly relax to a stationary profile after channel opening [20], [32], [33]. In this situation, the stationary approximation in Eq. 2 is justified. Below we will complement it with a simple description of temporal relaxation of 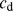 after channel closing.

We now summarize the static [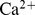] part of the modeling features for 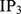
R clusters:

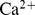
 released from a channel creates a nanodomain near the channel pore, which is spatially small but exhibits 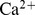 at very high concentration. An open channel is exposed directly to this nanodomain. Consequently, the 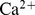 concentration governing further gating transitions of the open channel is large. We denote the corresponding concentration value by
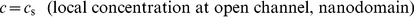
(3)and choose 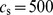 µM for our simulations [33].
- If a channel is closed, the local concentration at that channel originating from 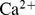 released by 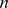 other open channels is described well by a linear relation [17]

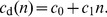
(4)This quantity provides a typical concentration within the cluster microdomain. If channels are open (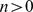), 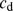 is much larger than the 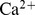 concentration a few *μ*m away from the cluster. But it is also much smaller than the local value 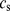 at the pore of an open channel. In [17] we concluded from detailed comparison with experimental results that the scale-separation is needed for agreement. We here use 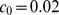 µM and 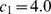 µM , where 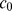 denotes the resting level concentration of 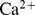 .

We will assume a cluster that consists of 20 channels. The values of 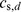 can be determined by two different methods. If one knows the full time-dependent evolution of the 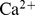 distribution during puffs, 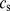 and 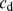 and their dependence on the number of open channels can be determined by averaging the local [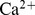] at all open channels or all closed channels, respectively [17], [20]. They can, however, also be introduced as phenomenological parameters. In [17] we have determined realistic values and have shown that this latter approach yields a very accurate description of experimentally observed puffs. The parameter value for the coupling concentration 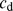 corresponds to a cluster with radius of around 300 nm. As will be seen below, this setup leads to typical numbers of open channels of around 5–10, consistent with experiments in neuroblastoma cells.

### Non-stationary microdomains and mobile buffers

A further important factor in 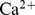 signaling is the role of 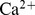 buffers. It is well-known that mobile buffers can strongly reduce the size of microdomains and thereby also decrease the amount of 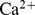 that reaches a target by diffusion. Here, however, we would like to focus on a property of buffers, which was much less studied so far, namely the influence of buffers on the microdomain collapse after channel closing, *i.e.*, the residual 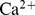 .

In [20] we have studied the effect of mobile and immobile buffers on microdomains. By detailed numerical simulations of three-dimensional reaction-diffusion equations we characterized the alteration of open channel domains by buffers. Most importantly, we found that mobile buffers reduce the amount of residual 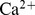 . This happens because mobile buffers may bind free 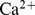 and support the transport of 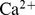 away from the microdomain. They, therefore, speed up the collapse of residual [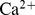] .

We also described in [20] which buffers can reduce the spatial extension of a domain around an open channel. It happens only if the buffer is mobile *and* has fast reaction kinetics. As described in [20] the fast mobile buffer plays a crucial role in reducing the coupling of channels within a cluster. Within our modeling approach this would result in a decrease of coupling constant 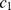 in Eq. 4 owing to the presence of fast mobile buffer. However, we are here interested in the role of slow mobile buffers, such as EGTA, which have little effect on the spatial extent of microdomain. Therefore, in the remainder of this paper, we neglect any changes of spatial appearance. We will therefore fix the coupling parameter 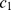 and exclusively study the effect of microdomain [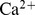] decay caused by slow mobile buffers.

It remains to cast the temporal evolution law for the 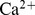 microdomain after changes in the open channel number. As has been described earlier, after opening of a channel, the equilibration of [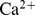] within the microdomain occurs very fast [20], [32]. Therefore, we assume instantaneous [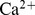] equilibration according to Eq. 4 after an increase in open channel number 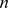. However, after channel closing, residual 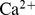 remains and we here use an approximation of this microdomain collapse with an exponential equilibration:
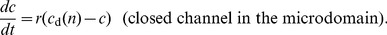
(5)Here 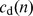 is given by Eq. 4. 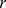 is the rate of equilibration. Obviously, this simple law does not reflect the full complexities in the microdomain collapse but it does here serve as the most simple model for qualitative analysis [20], [34].

### Summary of method

**Figure 4:**
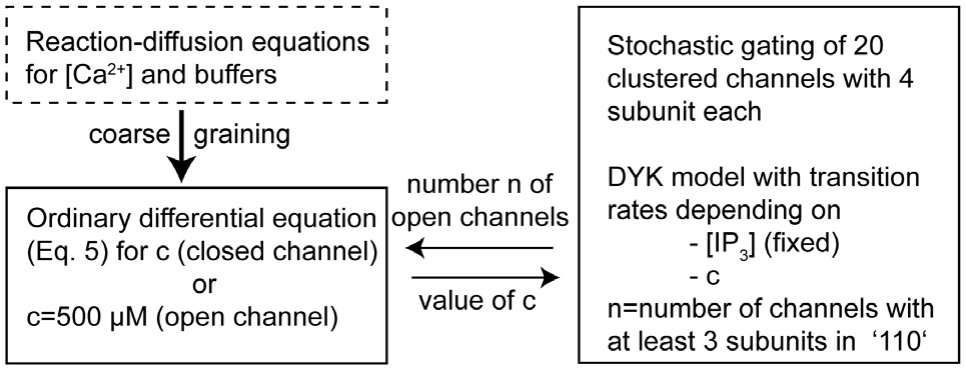
Chart of the basic modeling setup used in this study. A deterministic scheme for the 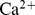 concentration is coupled to a stochastic description of channel gating (solid boxes). The state of 20 channels is simulated using a kinetic Monte-Carlo method. The number of open channels is determined at each instance of time. From the number of open channels one determines the concentration of 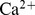 at open and closed channels using a fixed value or an ordinary differential equation, respectively. This reduced model is obtained by coarse-graining from direct numerical simulations of the full partial differential equations (dashed box). Parameters of the coarse-grained model depend on the physiological parameters such as channel distance and 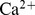 buffers.

A summarizing chart of our model is given in Fig. 4. In the following we simulate clusters of 20 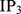
R channels. Each subunit of each channel undergoes stochastic transitions according to the scheme shown in Fig. 2B. We use a standard stochastic algorithm based on a small time-step 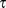. For each time step the algorithm determines for each subunit whether a state transitions occurs (for a description of the stochastic simulation method see Supporting Text S2).

The channel is considered open if at least three of its four subunits are in the state 110, otherwise it is considered closed. The 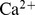 concentration variable 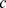 appearing in the scheme in Fig. 2B is a quantity that follows the (stochastic) number of open channels in the cluster. It is governed by our coarse-grained deterministic model and 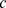 is thus not intrinsically stochastic. In each time step we determine the 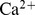 concentration from the number of open channels 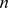 and by the two-scaled 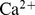 concentrations given by Eqs. 3–5. More specifically, for all subunits that belong to closed channels, 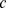 is given by the value in differential equation 5. Subunits that belong to open channels will be given 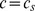 in scheme Fig. 2B.

Finally, the value 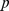 in scheme Fig. 2B is the 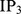 concentration. Our choice of [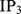] will be within a range of concentrations where non-trivial dynamics can be expected. Recent experimental estimation of [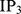] during 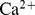 release have shown that values of [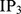] between 50 and 100 nM are optimal for the appearance of 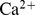 oscillations in COS-7 cells [35]. We will use values in the according range in the following discussions.

Parameters in the ordinary differential equation for 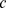 depend on the cellular physiology. For instance, the coupling constant 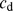 depends on the distance of channels and the presence of 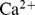 buffers. However, in the current study we only consider changes in the relaxation rate 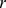. An increase of 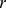 corresponds to a faster collapse of the 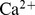 microdomain. Large 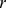 (here typically 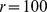 s^
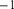
^) thus represents the case of EGTA-influenced release, while small 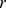 (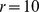 s^
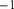
^) represents the normal, unimpeded case.

## Results

### Dependence of release dynamics on 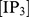


**Figure 5:**
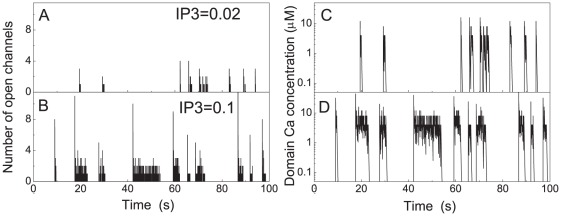
Evolution of open channel number (A,B) and microdomain 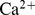 concentration 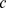 (C,D) for clusters with 20 channels and decay rate 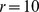 s^
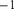
^. A and C show traces for [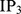] = 0.02 µM , B and D for [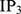] = 0.1 µM.

A first analysis concerns the behavior of release events under changes of [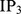] using a decay rate of 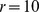 s^
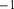
^. This small value of 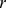 and the resulting slow temporal collapse of the 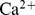 microdomain, can be viewed as a control “experiment” without slow mobile buffer. Fig. 5 shows the typical time evolution of the number of open channels and the microdomain concentration 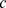 for two different values of 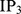 concentration. In both cases, well-defined release events (puffs or wave-like bursts) are apparent.

**Figure 6:**
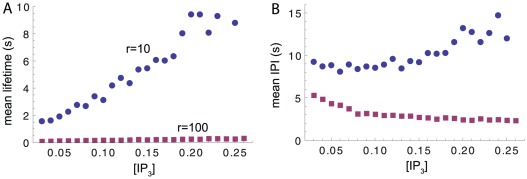
Dependence of mean lifetime (A) and mean interpuff interval (B) on the concentration of [ 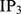] for two different values of decay rate 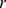. 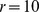
 s^
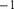
^ corresponds to long transients expected in absence of slow mobile buffer. Waves are experimentally found for large [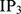] but not in the presence of slow mobile buffer.

To analyze their properties statistically, we now define a release event based on the evolution of the number of open channels. An event begins with the transition from 0 to 1 open channel and ends when the last channel closes, *i.e.*, when the number of channels is at zero again. However, to account for the noisy gating behavior sometimes observed in the wake of a release event, we consider the transition from 1 to 0 open channels not to be the end of the release event, if a re-opening of a channel occurs within a time span of 500 ms. The event is then considered terminated at the closing of the last channel if no further opening occurs within 500 ms. In Fig. 6 we characterize traces such as those in Fig. 5A,B accordingly for a range of values of [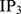] . Here we have evaluated the typical lifetime of release events as well as the duration between consecutive events (interpuff interval, IPI). Fig. 6 shows that for 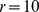 s^
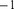
^ both mean lifetime and mean interpuff interval generally increase with [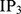] .

This result can be related to the transition from puffs to waves under increase of cytosolic 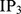 concentration. Within a stochastic theory of 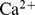 release, wave generation can be understood in the following way. Waves begin by the random opening of one or a few clusters [36]. The released calcium spreads to neighboring clusters and because of their 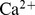 dependent probability to open, the initial event triggers a wave. Increase of 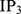 supports the propagation of release activity since the duration and/or amplitude of the local trigger event increases and the excitability of neighboring clusters can increase. In this sense our result in Figs. 6A principally allows to interpret the increase of lifetime as a transition from local to global release. When the [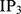] parameter is increased, the duration of the cluster release prolongs. Therefore, for large [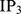] cluster cooperativity is likely, while for small [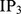] puffs are too short to allow propagation of activity. Here, however, we will not pursue this line of thought but instead focus on the effect of a change in decay rate 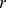.

### Dependence of release dynamics on decay rate

Fig. 6 also shows that the lifetime and IPI of release events strongly depends on the decay rate 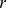. For large 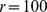 s^
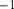
^ the mean lifetime is much smaller than for 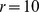 s^
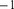
^. We will now consider this dependence and its origin in detail. The 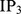 concentration will be set to 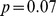 *μ*M in the following. This value is larger than 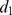, so that in rest state the 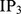
R subunits are normally occupied by IP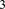 and reside in the upper plane of the DYK cube (Fig. 2B).

**Figure 7:**
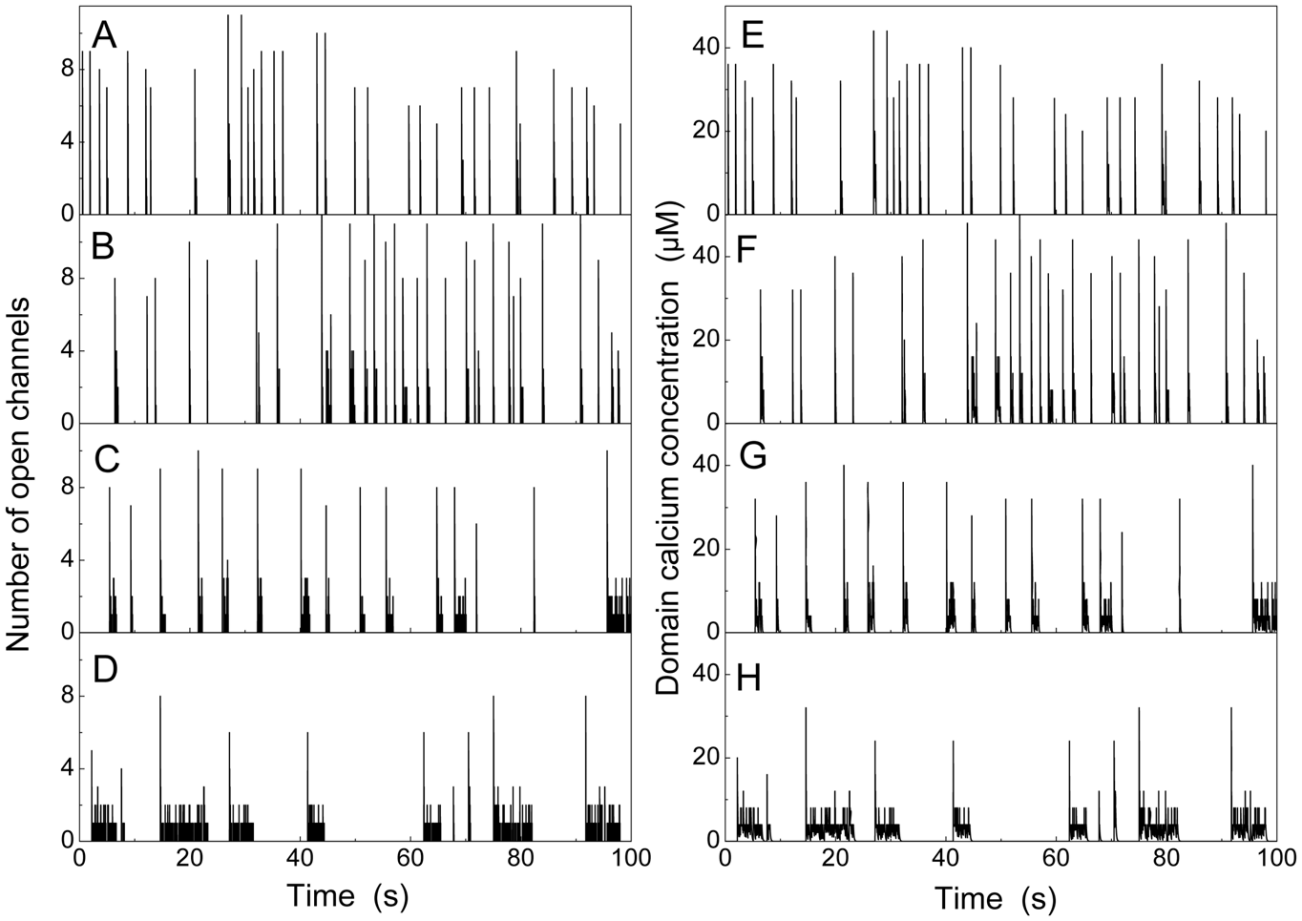
Evolution of open channel number (A–D) and microdomain 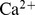 concentration 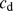 (E–H) for various decay rates 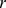. With decreasing 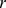 the trace shows longer and fewer events (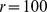 s^
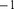
^ (A,E), 50 s^
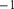
^ (B,F), 20 s^
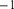
^ (C,G), 10 s^
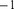
^ (D,H) at [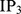] = 0.07 µM ).

Fig. 7 shows the typical evolution of open channel numbers for different values of the decay rate 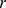. For large decay rate, 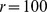 s^
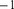
^ we find frequent short spikes that resemble the experimentally observed puffs lasting less than 1 second. However, for smaller 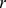, long events occur, which typically consist of one high initial spike and a burst of openings of decreasing amplitude (wave regime). Lifetime of wave-like release becomes increasingly longer as the decay rate decreases (Fig. 7).

**Figure 8:**
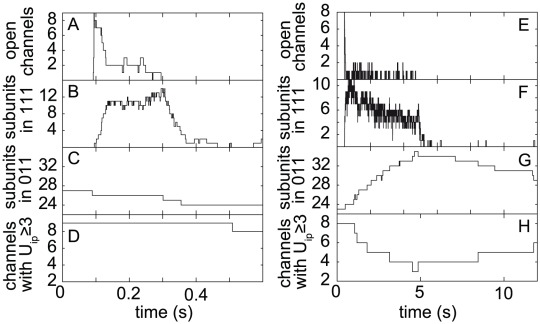
Analysis of a single puff or burst for fast 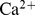 decay, 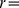
100 s^
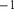
^ (left) and slow decay, 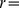
10 s^
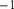
^ (right). Plots show the number of open channels (A,E), the number of subunits in the state 111 (B,F) and 011 (C,G) and the number of channels that have at least three subunits with 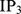 bound (D,H). Here 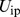 denotes the number of subunits of a channel, which have 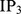 bound.

We will next analyze the behavior of channel gating that leads to the different dynamics of puffs and waves. Starting with the case of puffs (fast microdomain collapse), Fig. 8A shows the evolution of the number of open channels and Figs. 8B–D present the three quantities characterizing the inhibition and refractory behavior. Figs. 8B and C show the numbers of subunits in the particular inhibited states 111 and 011, respectively. The state 111 is obtained by direct transition from the open state, 110, while the local 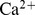 is large. Fig. 8B clearly shows the fast onset of inhibition, which leads to the closing of all channels within about 200 ms. Because of the fast time scale of inhibition, not only is there a fast onset of inhibition, but, as seen in Fig. 8B, there is also only a short refractory time. The latter is caused by a fast rate of de-inhibition, 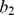, which is around 2 s^
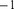
^. This implies that the channels can be re-activated almost immediately after the termination of the last puff. However, because of the fast decay of 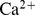 concentration for large 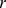, calcium ions are normally too dilute to cause immediate re-openings.

**Figure 9:**
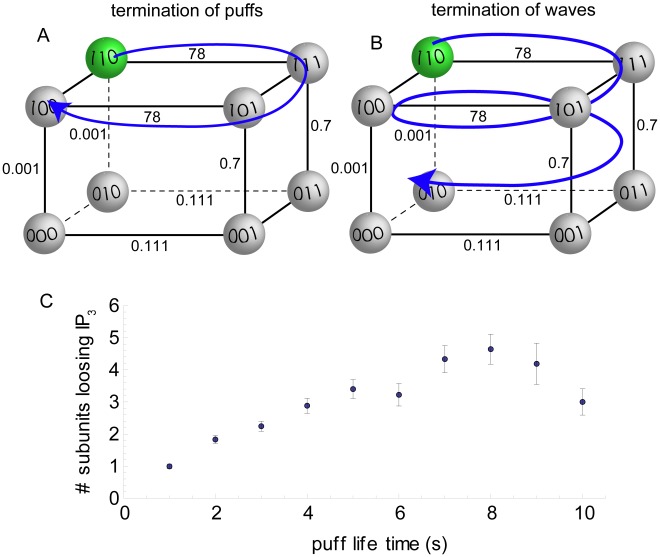
A and B illustrate the dynamics of subunits during a puffs (A) and burst (B). During short release (puff) only the upper plane of the DYK cube is populated by subunits of opening/closing channels (A). However,, the more frequent the channels open, subunits in state 111 dissociate 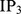 and “cycle” down to the lower plane of the cube (B). Numbers show the dissociation constants of our model in µM . The loss of 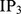 occurs only for inhibited subunits, since only for inhibited subunits is [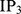] smaller than the dissociation constant (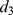 = 0.7 µM ). (C) shows the number of subunits that loose 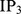 during the course of a single puff or burst of puffs. For short puffs, few subunits dissociate 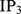, while for long-lasting bursts up to 5 subunits loose 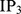. The corresponding channels typically loose the ability to open. The data is taken from a single, long run with [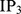] = 0.07 µM and 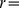
10 s^
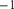
^.

We conclude that for (short) puffs the dynamics involves 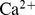 activation and 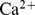 inhibition of subunits. This result confirms earlier models of 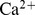 dynamics, which relate the termination of 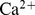 puffs to the binding of 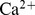 to inhibiting binding site. This dynamics therefore does not need and does not incorporate the third component of the DYK model, which is the binding or unbinding of 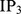 . Fig. 8C shows that the number of subunits in state 011 (those subunits that have not bound 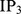 but have 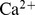 bound to the activating and inhibiting sites) does not change much during a puff. Fig. 8D shows the number of channels, 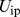, that have bound 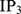 to three or four subunits. Note that 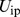 in is the number of channels that are principally available for opening during a puff. Typically only a fraction of the 20 channels is available for opening (this effect will be discussed below.) Thus, Fig. 8D indicates no changes in 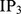 occupation during a puff. Taken together, our observations indicate that a typical trajectory of the subunits during puffs is a cycle that involves 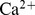 activation and inhibition only. It thus only involves the upper horizontal plane of the DYK cube (see Fig. 9A).

We now discuss the behavior during the wave-like events occurring for smaller decay rate 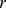 (persistent 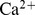 microdomains). Similarly to the case of short puffs, rapid inhibition occurs, which involves units transferring to the 111 state (Fig. 8F). However, because of the large transient microdomain [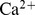] , immediately after termination of an opening and restoration of subunits to the de-inhibited state, a further opening of channels occurs. This behavior leads to the perpetual cycling of some of the subunits around the upper plane of the DYK cube, explaining the occurrence of long bursts of openings shown in Fig. 8E.

One may think that the existence of longer signals for persistent 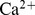 microdomains is simply a consequence of subunits cycling the activation/inhibition loop. However, we will now explain that the four states of the upper DYK cube are not sufficient to obtain the well-defined bursts shown in Fig. 8E. This relates to the fact that subunits cycling along the upper loop need a further mechanism if they are to finally terminate the 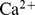 release. Since inhibition is already part of the cycling that builds the bursts, another mechanism is needed to end the bursts. Surprisingly, our model suggests that the dissociation of 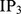 from subunits provides this mechanism.

First, Fig. 8G shows that during the course of a wave, more subunits accumulate in the state 011. The number of subunits in this state increases for this particular burst from 22 to 34, that is 12 subunits dissociate 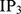 during the burst. Note that the rates of 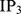 binding/unbinding are smaller than those of the other processes involved during a puff (see Supporting Text S1). Further, many of the channels have only three of the subunits bound to 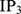 , so that a further dissociated subunit means that the channel is not available for opening. Accordingly, the number of available channels drops from 8 to 3 during the burst event (Fig. 8H).

We found that the subunits undergo the 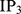 dissociation while being in the state 111. The mechanism of 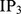 dissociation therefore depends on the time that a subunit spends in the 111 state. This means that the 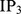 dissociation increases with the duration of 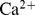 release. Fig. 9C shows that 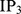 dissociation indeed occurs for long release events. It thus constitutes a second inactivation process that is needed to terminate the wave-like release. Fig. 9B illustrates the mechanism, which involves gradual 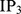 dissociation of subunits while cycling the 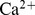 activation/inhibition loop.

The 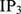 unbinding mechanism relies on the fact that the dissociation constants of 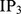 binding are very different for subunits with and without 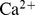 bound to the inhibiting site. Stimulating levels of 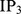 concentration ( *i.e.*, 0.07 µM in our simulations) are well above the value 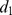 (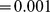 µM ), so that many subunits have bound 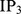 . However, inhibited subunits possess a much higher dissociation constant 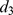 (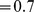 µM ), such that loss of 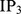 can occur while subunits populate the inhibited states. Therefore, the termination of waves due to 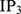 dissociation is possible only when the 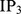 binding depends on inhibition. This leads to the conclusion that wave termination is related to the shift of peak open probability with 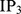 concentration (see Fig. 2B).

### Behavior of lifetime and interpuff interval and comparison to experiments

**Figure 10:**
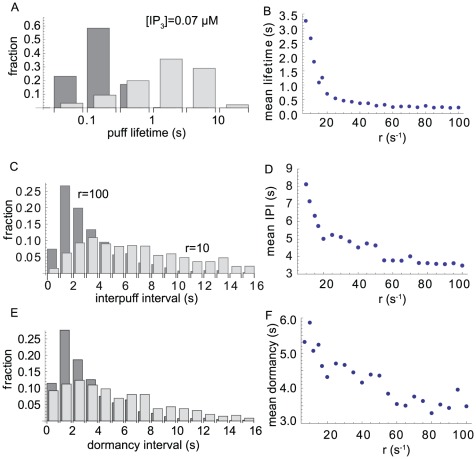
(A) Lifetime distribution for 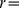
10 s^
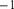
^ (light gray) and 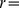
100 s^
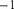
^ (dark gray). (B) The mean life time decreases with increasing decay rate 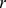. (C) IPI distribution for 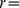
10 s^
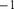
^ and 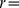
100 s^
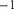
^. (D) The mean interpuff interval decreases with increasing decay rate 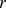. (E) and (F) show the dormancy interval, *i.e.*, the time between puffs, during which no channel is open. (All data for 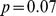 µM.)

The effect described above lead to strong changes in the statistical properties of release events depending on the decay rate 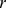. This concerns first of all the distribution of release lifetime. For fast collapse of the 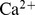 microdomain, most events are of short duration. As an example, Fig. 10A shows the lifetime distribution for 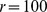 s^
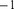
^ (dark gray bars). Most puffs in this case last between 100 and 300 ms. If, however, the 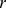 value is substantially lowered, most events last considerably longer than 1 s (light gray bars). Clearly, this behavior follows from the slow decay of microdomain 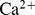 values and the related re-opening of channels.

Fig. 10B shows the dependence of average event lifetime on 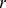. In the chosen range of 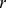 the event lifetime varies over one order of magnitude, which is what was observed in [12],[31]. Our results resemble well the experimental findings for dependence of 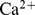 release spikes if 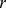 is associated to the level of buffer concentration.

Interpuff intervals (IPI) have been experimentally determined as well. For puffs of small amplitude, Fraiman *et al.* found a distribution of IPIs peaked between 1 and 2 seconds and strongly decaying for larger intervals [37]. As can be seen in Fig. 10C, simulations of our model for 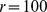 s^
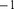
^ yield a similar distribution with rare occurrence of IPIs with more than a few seconds (dark gray bars). If 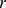 is much smaller than this value, the distribution is much flatter. Correspondingly, the smaller the 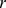 the larger is the mean IPI (Fig. 10D).

The mean IPIs found in our simulations agree quantitatively with experimental values. In SH-SY5Y cells loaded with EGTA, puffs occur with a frequency of about 0.23 Hz, roughly corresponding to an IPI of four seconds [31]. In the absence of EGTA, the experimental IPIs increase to about six seconds, again agreeing with our mean IPIs for small 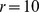 s^
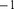
^. These numbers demonstrate the realistic representation of experimental results in [31] by our modeling approach.

In view of the mechanism proposed in this paper, the increase of IPI for small 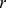 appears to be counter-intuitive. If residual 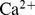 is large, one would naively expect that a higher activation probability and thus an earlier appearance of the next wave results. Therefore, for small 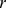 one could expect shorter IPIs. Here, however, we find for small 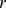 that the next wave occurs typically several seconds later. In fact, this delay is not the result of simply a longer lifetime of release events. Figs. 10E,F present the time of dormancy between two consecutive release events, *i.e.*, the length of intervals in which no channel is open. The increase of dormancy interval for persistent residual microdomains (small 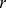) indicates the presence of a refractory mechanism that suppresses the probability of wave generation for a few seconds. We briefly would like to discuss the origin of this behavior.

We first remind that for large 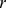 a very short refractory period after the termination of a puff was noticed (see Fig. 8B). The refractory period is the time where channels are inhibited or otherwise not available for re-opening. This time is of the order of at most a few hundreds ms. This means that the refractory period cannot account for the mean IPI for large 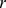, which is at around 4 s. Instead, most of the IPI for large 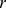 is related to a waiting time for a first “trigger” channel to open [38].

The small 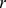 case, however, provides a different scenario. Clearly, the large number of 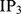 unbinding subunits decreases the probability of re-opening. The loss of 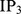 thus amounts to a kind of refractory period. The chance of triggering a wave is much smaller after long-lasting events where many 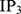 molecules are lost (Fig. 9C), mainly because the number of channels available for opening is smaller. Only after this refractory period, channels have re-bound the lost 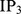 and regain their potential to trigger a wave. It is interesting to note that this 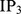 refractoriness should generally reduce 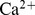 excitability. It may therefore provide a new mechanistic explanation for the smaller excitability of cells for the first few seconds after waves [36].

## Discussion

The spatio-temporal patterns of 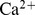 signals are known to depend on many factors, including the 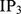 concentration and the presence of buffer proteins. We have here discussed the role of “hidden” quantities in the shaping of 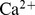 signals and argued that they may serve as mediators of signal modulation. We have shown that their dynamics can explain features of 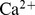 puffs and waves in a consistent way.

Most importantly, our analysis reveals that changes in the collapse of 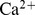 microdomains can have a profound effect on release duration. In this paper, we have represented this collapse of 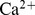 microdomain by the rate 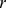, which describes the decay of residual 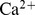 in the microdomain after closing of channels. The most significant result is that for slow collapse (small 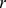) one generally finds long release reminiscent to temporal evolution during global waves, while for fast collapse much shorter events – puffs – appear. For slow collapse, residual free 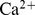 re-activates channels by binding to their activating binding sites soon after channel closing. Often, re-activation leads to re-opening of channels because the refractory time of open channels is relatively short, *i.e.*, inhibited subunits unbind 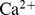 from their inhibited sites shortly after the channel has closed and are therefore available for re-opening. This property distinguishes the 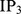 - 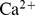 system from other excitable systems where refractoriness lasts much longer than the actual spike [39].

### Termination of puffs and waves

Since the durations of puffs and waves are so different, it is natural to expect that there should be different processes responsible for the termination of release. Many experimental and modeling studies have proposed that puffs are terminated by 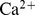 -inhibition. Our earlier results [17] and the analysis presented here also support the idea that puffs are terminated by 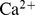 binding to inhibiting sites. An unexpected outcome of our work is that for “long puffs” (or waves) termination occurs largely by way of 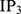 -unbinding. This effect is based on the dependence of 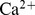 dissociation constants on 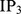 . The special form of the dependence, as well as the related shift in the peak location of open probability, is an often neglected trait of 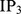 receptor gating. 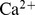 inhibition and 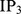 dissociation thus present dual mechanisms that not only provide a surprising answer to the long-standing puzzle of release termination, but also assign a function to the control of 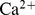 inhibition by 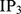 . Our results highlight the meaning of complex 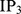
R gating models such as the DYK model and require that future studies need not oversimplify 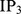
R gating schemes.

### Buffers and the puff-wave transition

The effect that we describe provides a novel explanation of the action of EGTA buffer on 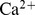 release. Experiments with various exogenous buffers have clearly demonstrated the strong effect of buffers on dynamics of 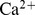 release [12], [40]. Dargan et al. [12], [41] studied how the 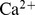 signal depends on buffers injected into *Xenopus* oocytes. In these experiments, the amount of 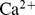 in the cytosol after stimulation by 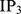 was measured by fluorescence recordings using an additional dye buffer. For EGTA buffer the response to the 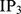 stimulation was sharply shortened compared to the release in untreated cells. In contrast, BAPTA-injection leads to a more homogeneous and prolonged release.

By relating large values of 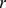 with the action of EGTA on calcium domains our results imply a shortening effect of EGTA on release of calcium. It is important to note that the release duration here refers to the proper open state of channels in a cluster and not to indirect effects due to a dye-buffer competition, which may additionally shorten traces of puffs in cells loaded with EGTA [12], [42]. To our knowledge, our work is the first theoretical study to show the consequences of EGTA and similar slow buffers on channel gating dynamics within a cluster.

How realistic is our assumption that the presence of EGTA speeds up the collapse of 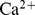 microdomains? Earlier work on full reaction-diffusion equations for buffer and 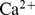 has shown that the collapse of free 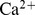 concentration at the channel pore after closing of the channel strongly depends on the mobile buffer present in the system [20]. Our detailed three-dimensional simulations have shown, for instance, that in the presence of EGTA a free 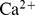 concentration of 0.5 *μ*M is reached after less than 1 ms, while in the absence of EGTA it takes about 6 ms. This observation serves as motivation for the simplified decay dynamics for free 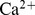 concentration in our model.

Having argued that the effect of EGTA is to accelerate the collapse of 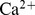 microdomain, it remains to discuss why, experimentally, the presence of BAPTA does not lead to puffs and shortening of release. The difference between the cases of EGTA and BAPTA can be attributed to the different kinetic rates of 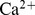 binding/unbinding. EGTA is a buffer that binds 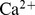 relatively slow, while BAPTA reacts around 100 times faster. This implies that BAPTA can interupt the communication between channels during a puff [20], while EGTA is too slow to bind substantial amounts of 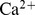 during a puff. In other words, BAPTA reduces the peak levels of microdomain [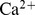] in Fig. 1 *and* residual [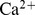] , while EGTA only diminishes residual [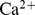] . We therefore speculate that the fast intra-cluster action of BAPTA leads to a very different dynamics, for instance by reducing the initial phase of a puff or reducing inhibition. Detailed analysis of this problem will be performed in the future.

With respect to physiological conditions *in vivo*, our results suggest that the duration of 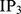 -evoked 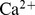 signals is a highly variable quantity. Experimentally the lifetime depends not only on 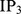 concentration and buffer content but was also shown to be sensitive for instance to changes in temperature [43]. The mobile buffer, however, appears to be special in that it allows control not only of the duration of release events but also of channel cooperativity. The concentration of cell specific buffer such as the 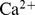 binding protein parvalbumin is one example showing the importance for tuning the duration of local 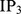 -evoked 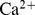 signals. Parvalbumin is a slow 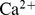 binding protein, which is known to have an important physiological role in muscle and neuronal cells. John *et al.* have shown that parvalbumin, similar to EGTA, inhibits repetitive 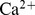 waves and evokes release at discrete release sites [22]. Buffer concentration determines the sensitivity and cooperativity of 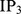 action and confers a threshold for the ability of the cell to transition from a local to a global mode of 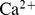 signaling. It is therefore possible that cell-specific expression of parvalbumin and potentially other buffers may serve to shape intracellular 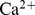 puff and wave signals for specific physiological roles by the mechanism described in this paper.

## Supporting Information

Text S1 Method and results of the single channel data fitting.
(PDF)

Text S2 Description of stochastic simulation method.
(PDF)
