# Supplementary material for: Termination of Ca2+ Release for Clustered IP3R Channels
Source: PLoS Comput Biol. 2012 May 31;8(5):e1002485. doi: 10.1371/journal.pcbi.1002485 (PMC3364945; doi:10.1371/journal.pcbi.1002485)
Supplement: Text S1 — Method and results of the single channel data fitting. (PDF) [file pcbi.1002485.s001.pdf]

Text S1: Fitting the single channel data

The scheme shown in Fig. 2B represents the DeYoung-Keizer model [1]. In equilibrium situations one fixes Ca^{2+} and IP_3 concentrations, c and p, respectively. The equilibrium behavior can be discussed by means of statistical measures such as channel open probability, mean open time and mean close time. The probability of an IP_3R subunit being in state (ijk) is denoted by P_{ijk} with $\sum P_{ijk} = 1$. By mass action kinetics, the equations describing the subunit dynamics are

$$\frac{\mathrm{d}P}{\mathrm{d}t} = PQ, \tag{1}$$

where Q is the generator matrix of transition rates and P is the vector of probability of subunits. Mathematically, the equilibrium state is defined as $\mathrm{d}P/\mathrm{d}t = 0$. The equilibrium vector w satisfies $wQ = 0$ according to the transition matrix theory [2, 3]. Detailed balance is imposed so that we can solve for the vector w. This is done by calculating the probabilities in terms of their probabilities relative to state (000) along the shortest binding/unbinding path. These unnormalized probabilities are denoted as q_{ijk} with $q_{000} = 1$. As an example, the equilibrium probability q_{110} of state (110) relative to that of state (000) is the product of forward to backward rates along any of the shortest paths connecting (000) to (110) given as

$$q_{110} = \frac{pc}{d_1 d_5}. \tag{2}$$

Then the normalized equilibrium probability for state (ijk) is $w_{ijk} = q_{ijk}/Z$ where Z is the normalization factor defined by $Z = \sum q_{ijk}$ and given as

$$Z = 1 + \frac{c}{d_4} + \frac{c}{d_5} + \frac{c^2}{d_4 d_5} + \frac{p}{d_1} + \frac{pc}{d_1 d_2} + \frac{pc}{d_1 d_5} + \frac{pc^2}{d_1 d_2 d_5}. \tag{3}$$

Thus, the normalized equilibrium probability for state (110) is as follows:

$$w_{110} = \frac{pc}{d_1 d_5 Z}. \tag{4}$$

For the DYK model, the channel opens when three out of four subunits are in state (110), so the channel open probability is written as

$$P_{\mathrm{O}} = P_{4\mathrm{O}} + P_{3\mathrm{O}} = w_{110}^4 + 4w_{110}^3(1 - w_{110}) \tag{5}$$

with $P_{4\mathrm{O}} = w_{110}^4$ and $P_{3\mathrm{O}} = 4w_{110}^3(1 - w_{110})$. Because channel states (110, 110, 110, not 110) are the only open states that connect to closed channel states by any one of three (110) states changing to the other three states, *i.e.*, 100, 010, or 111 with rate b_5, b_1, or $a_2 c$, we can directly write the equilibrium probability flux between open and close states as follows:

$$J = 3P_{3\mathrm{O}}(b_1 + b_5 + a_2 c). \tag{6}$$

The mean open and close times are then given by

$$\tau_{\mathrm{O}} = \frac{P_{\mathrm{O}}}{J},\ \tau_{\mathrm{C}} = \frac{1 - P_{\mathrm{O}}}{J}. \tag{7}$$

Solid lines in Fig. 2B show the open probability for a DYK model where parameters were fitted against patch clamp data. Parameters d_i where obtained by fitting equilibrium binding fractions to experimental open probabilities for various values of c and p.

The remaining parameters a_i are less clear from the experiments and for some of them a wide range of values have been used in the literature. Generally one obtains them by comparison of model predictions of mean open and closed times to experiments [3]. Furthermore, parameters can be estimated by comparison to data from puffs. For instance, the parameter a_5 can be fitted to the rate of channel recruitment during puff initiation [4].

The only substantial deviation of our parameters from other studies occurs in the time-scale of IP_3 binding and unbinding. As shown in our paper, unbinding of IP_3 determines the duration of life-time and IPI of long-release events (waves). The time-scale should therefore be of the order of seconds. Accordingly, unbinding of IP_3 in the inhibited state is governed by b_3 and is in our model set to 0.14 s^{-1} (see Appendix of main text). Note that this rate is difficult to assess directly in single channel patch clamp experiments since it involves keeping the channel initially both inhibited by large [Ca^{2+}] and bound to IP_3 [5].

The following table lists the parameters used for the model in this paper and their values. The on-rates a_i, off-rates b_i and the dissociation constants d_i are related by $d_i = b_i/a_i$.

**parameter**	**symbol**	**value**	**unit**
IP_3-binding			
without inh. Ca^{2+}	a_1	0.2	$(\mu M\ s)^{-1}$
	d_1	0.001	μM
with inh. Ca^{2+}	a_3	0.2	$(\mu M\ s)^{-1}$
	d_3	0.7	μM
activating Ca^{2+}			
	a_5	100	$(\mu M\ s)^{-1}$
	d_5	0.25	μM
inhibiting Ca^{2+}			
with IP_3	a_2	0.02	$(\mu M\ s)^{-1}$
	d_2	78	μM
without IP_3	a_4	0.1	$(\mu M\ s)^{-1}$
	d_4	0.111	μM
IP_3 concentration	p	0.07	μM

References

1. DeYoung G, Keizer J (1992) A single-pool inositol 1,4,5-trisphosphate-receptor-based model for agonist-stimulated oscillations in Ca^{2+} concentration. ProcNatlAcadSci USA 89: 9895–9899.

2. Bruno W, Yang J, Pearson J (2005) Using independent open-to-closed transitions to simplify aggregated Markov models of ion channel gating kinetics. Proceedings of the National Academy of Sciences of the United States of America 102: 6326.

3. Shuai J, Yang D, Pearson J, Rüdiger S (2009) An investigation of models of the IPR channel in Xenopus oocyte. Chaos 19: 037105.

4. Smith I, Parker I (2009) Imaging the quantal substructure of single IP3R channel activity during Ca2+ puffs in intact mammalian cells. Proc Natl Acad Sci USA 106: 6404.

5. Mak D, Pearson J, Loong K, Datta S, Fernández-Mongil M, et al. (2007) Rapid ligand-regulated gating kinetics of single inositol 1, 4, 5-trisphosphate receptor ca2+ release channels. EMBO reports 8: 1044–1051.
