# Supplementary material for: Termination of Ca2+ Release for Clustered IP3R Channels
Source: PLoS Comput Biol. 2012 May 31;8(5):e1002485. doi: 10.1371/journal.pcbi.1002485 (PMC3364945; doi:10.1371/journal.pcbi.1002485)
Supplement: Text S2 — Description of stochastic simulation method. (PDF) [file pcbi.1002485.s002.pdf]

Text S2: Stochastic simulation method

In non-equilibrium simulations we have coupled the gating transitions to the evolution of Ca^{2+} concentration c. At each time step we first determine the number of open channels n. A channel is considered open if at least three of its four subunits are in the open state. Using Eqs. 3-5 we then compute the Ca^{2+} concentrations at open ($c = c_s$) and closed channels (Eqs. 4 and 5). For subunits that belong to open or closed channels, the respective c values are then inserted into the transition rates to obtain the propensities a_i for each subunit transition. For instance, a subunit in the state 110 that belongs to an open channel aquires the propensity $a_i = a_2 c = a_2 c_s$ for a transition to the inhibited state 111. As simple implementation of the stochastic algorithm one computes products of a_i and a small time step τ (=10^{-6} s in our simulations), which provides the probability that the transition occurs in the given time step. If $a_i \tau$ is larger then a random number drawn from a uniform distribution in $[0, 1]$ the transition is executed.
